# Five-Year Olds, but Not Chimpanzees, Attempt to Manage Their Reputations

**DOI:** 10.1371/journal.pone.0048433

**Published:** 2012-10-31

**Authors:** Jan M. Engelmann, Esther Herrmann, Michael Tomasello

**Affiliations:** Max Planck Institute for Evolutionary Anthropology, Leipzig, Germany; Ecole Normale Supérieure, France

## Abstract

Virtually all theories of the evolution of cooperation require that cooperators find ways to interact with one another selectively, to the exclusion of cheaters. This means that individuals must make reputational judgments about others as cooperators, based on either direct or indirect evidence. Humans, and possibly other species, add another component to the process: they know that they are being judged by others, and so they adjust their behavior in order to affect those judgments – so-called impression management. Here, we show for the first time that already preschool children engage in such behavior. In an experimental study, 5-year-old human children share more and steal less when they are being watched by a peer than when they are alone. In contrast, chimpanzees behave the same whether they are being watched by a groupmate or not. This species difference suggests that humans' concern for their own self-reputation, and their tendency to manage the impression they are making on others, may be unique to humans among primates.

## Introduction

A key mechanism for maintaining cooperation in social groups is reputation [1], [2]. Thus, many animal species engage in so-called partner choice, in which individuals known to be cooperative are favored in various social activities, and those known to be non-cooperative are shunned or avoided [3]. Being a good cooperator thus pays, and being a poor cooperator costs.

Among primates, great apes have been shown to make reputational judgments and partner choices of this kind. For example, Melis, Hare, & Tomasello [4] gave individual chimpanzees a choice of partners for a mutualistic collaborative task. They preferentially chose individuals whom they knew from direct experience to be good collaborators over those whom they knew from direct experience to be poor collaborators. Studies in which great apes observe interactions (between humans) from a third-party stance have yielded mixed results, but with at least some evidence for reputational judgments resulting in a preference for cooperators [5]–[7].

Humans of course make reputational judgments of cooperativeness all the time, but, in addition, they know that they themselves are often being judged, and so they have a concern for what might be called self-reputation. Given this knowledge and concern, humans often engage in what the sociologist Goffman [8] calls impression management (or self-presentation), acting so as to affect the reputational judgments of others toward the self. A concern for self-reputation and active attempts at impression management go beyond partner choice in which the individual being favored or shunned by others may not know that this process is going on and so make no attempts to control it. A number of experimental studies have demonstrated that human adults know when others are watching (indeed, they are even sensitive to pictures of eyes on the wall; [9], [10], and that they adjust their behavior accordingly (e.g. [2], [11], [12].

Human infants make something like reputational judgments – the process is typically called social evaluation – from as young as 6 months of age. Thus, Hamlin and colleagues [13] found that young infants preferred to interact with a puppet who had helped, rather than hindered, a third-party. But the age at which children become concerned with self-reputation and engage in active acts of impression management is not known. Virtually all studies of self-reputation are interview studies with school-age children in which participants have to linguistically formulate their concerns. For example, Aloise-Young [14] asked 6-year old children to give verbal self-descriptions to maximize their chances of subsequently being picked as a partner in a game. Similarly, Banerjee, Bennett, and Luke [15] asked children to verbally explain the self-reputational consequences of various rule violations.

Using these methods, positive results have been reported only for children 8 years of age or older. Banerjee [16] argues that the problem is likely motivational; that is, while 5-year-old children possess the necessary cognitive prerequisites for self-presentational behavior, they lack a concern for being socially evaluated (which emerges only during the primary school years). But it is also possible that preschoolers simply do not possess the linguistic skills and/or the self-awareness that would enable them to clearly articulate their concerns for self-reputation and self-presentational strategies. Supportive of this possibility, Piazza, Bering, and Ingram [17] found that 6-year olds behave more prosocially in the presence of an imaginary person than they do when they are in an unobserved condition.

In a first study, therefore, we assessed 5-year-old children's concerns for self-reputation – with special reference to cooperative behaviors – by observing them in two situations: helping and stealing. In some cases they were observed by a novel peer and in other cases they were alone. If children this young are concerned with their self-reputation for cooperation, we would expect them to help more and steal less when being observed. Importantly, the use of a novel peer observer instead of a familiar peer observer or an adult observer enabled us to rule out explanations based on familiarity, on the one hand, and authority and/or fear of punishment, on the other. In addition, recipients were absent and anonymous, so that any observed effects of condition could not be interpreted as due to interpersonal relationships or concerns about reciprocity [18]. We chose 5-year-olds as subjects as previous research has shown that it is at this age that children first engage in a central cognitive prerequisite of self-reputational behavior: second-order mental reasoning [19], [20] of the form “I am thinking about what you are thinking about me” [16].

To provide an evolutionary perspective on our results, we observed humans closest living relatives, chimpanzees, in a similar set of experimental situations. Although chimpanzees have been observed to produce food-associated calls differentially depending on which conspecifics are nearby – so-called “audience effects” [21] – these are not concerned with a reputation for cooperation and do not involve any impression management strategies. Based on our personal experiences with chimpanzees, we had a clear expectation that chimpanzees would not help more when being observed than when alone. But given that chimpanzees do sometimes engage in dominance displays, seemingly to impress others with their power [22], we thought it might be possible that they would steal more often when being observed, the opposite effect from that expected from children.

## Study 1: Children

### Method

#### Ethics Statement

The presented study was non-invasive and strictly adhered to the legal requirements of the country in which it was conducted. The study was approved by the Max Planck Institute for Evolutionary Anthropology Ethics Committee (members of the committee are Prof. M. Tomasello, head of the child lab Katharina Haberl, and research assistant Jana Jurkat). The full procedure of the study was covered by the committee's approval. Informed written consent was obtained from all the parents of the children who participated in this study.

#### Participants

We tested ninety-six 5-year-old children (*M* = 59 months 12 days; range  = 57 months and 5 days to 62 months and 27 days). 24 subjects (12 girls, 12 boys) participated in each of the four conditions (helping observed/helping unobserved; stealing observed/ stealing unobserved). Children were randomly assigned to one of the four conditions. All subjects were paired with same-sex observers (*M* = 71 months 8 days; range  = 69 months 7 days to 74 months 26 days). Subjects and observers attended the same day-care center but different groups within the center.

#### Materials

Two identical sticker sheets (sheet 1 and sheet 2) were positioned on a table at all times throughout the experiment. Five symbols had been drawn on every sticker sheet: a star, a small heart, a large heart, a smiley and a ladybird. During the experiments, children were handed the appropriate stickers in an envelope to conduct the respective tasks.

#### Procedure and Design

Each child participated in both a warm-up phase as well as a test phase. The warm-up phase was identical for all conditions. After the warm-up phase, each child participated in one of the four conditions: stealing unobserved, stealing observed, helping unobserved, or helping observed. In all four conditions, subjects engaged in the same sticker game with the aim of placing the appropriate stickers in all of the 5 predefined symbols. Also in all conditions, sticker sheet 1, intended for the subject, as well as sticker sheet 2, intended for another, anonymous participant, were placed at 20 cm distance on the table. Each participant underwent three trials in the respective condition. Testing took place in a quiet room of the day-care center in a single session.

Before the test phase, each subject engaged in a warm-up phase. This phase was included in order to ensure that subjects had understood the game. The warm-up game consisted of a smaller version of the test phase sticker game. Subjects had to place 3 stickers on the appropriate symbols on a sticker sheet within 90 seconds. All 96 children were successful on the first attempt, thus showing a strong understanding of the game.

**Figure 1:**
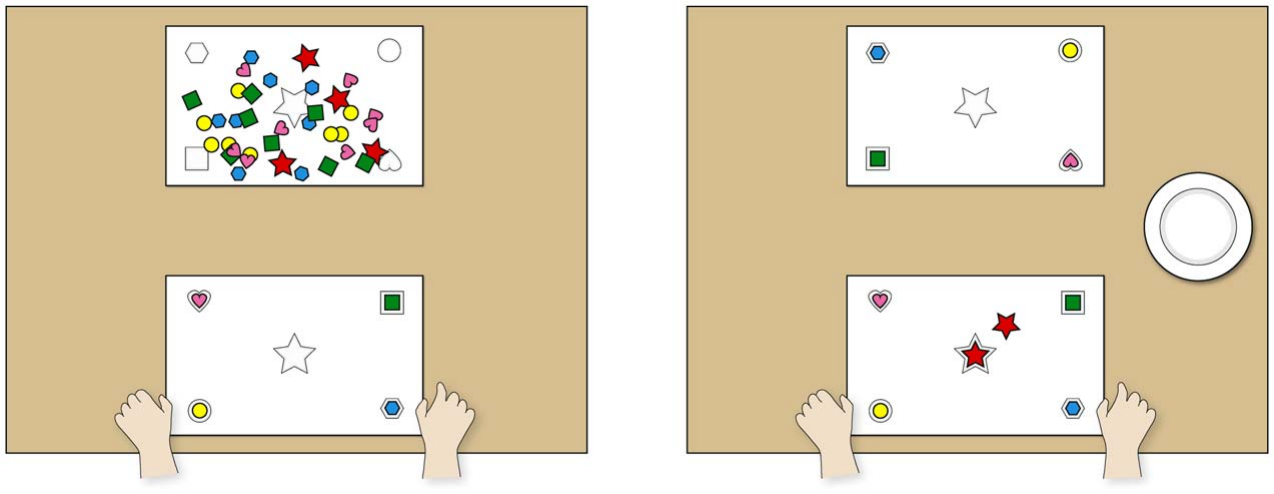
Setup of the child study. Illustration of the experimental setup for children, viewed from the subject's perspective. In the stealing task (left) subjects could only finish their sticker sheet if they took a star-shaped sticker from sticker sheet 2, destined for an anonymous next participant. In the helping task (right) subjects received one additional sticker. They had the option of either leaving the sticker for the next participant or taking it home.

For the stealing task, 5 copies of each of the 5 stickers were placed on sticker sheet 2 prior to the experiment. Once subjects had entered the room, they were told that sticker sheet 2 plus its stickers were intended for another child who would play the game later on. Then they received their own stickers from a separate envelope. However, subjects received only 4 stickers in their envelope. Thus, subjects were missing one sticker (star-shaped) to complete the given task. Experimenter 1 then left the room. After the child had placed all 4 stickers on her sticker sheet, the experimenter waited for 40 seconds and then reentered the room (Figure 1).

For the helping task, both sticker sheets were empty upon the subject's entrance. The experimenter then placed the sticker contents from one envelope on sheet 2 and handed the stickers from a second envelope to the child. The centrally located, star-shaped sticker was absent from sheet 2. The child was then told that if they got an additional sticker, they could either place it on sheet 2 for the next participant or keep it for themselves (by placing it on a small plate on the table as well). Order of the presentation of these options was counterbalanced across trials within subjects. After these instructions, Experimenter 1 left the room. Once the child had made a choice, he reentered the room (Figure 1).

For both of these tasks, subjects were alone in the testing room in the unobserved conditions. In the observed conditions, an observer was brought into the room prior to the start of the test phase by Experimenter 2 and placed on a chair at 1.5 meters from the subject. Observers were children from the same day-care center but belonged to different groups than the subjects. Prior to entering the testing room, observers were told by Experimenter 2 to closely watch the subjects. Experimenter 1 told subjects that they would be in the room with another child. To eliminate communication between observer and subject during the test phase, observers listened to an audiobook while watching the subject play (in addition, they were instructed not to engage in conversation with each other).

#### Coding and dependent measure

All trials were videotaped with 2 cameras and coded by the first author. A research assistant, who was unaware of the study design and hypothesis, independently coded 25% of all trials. Number of stealing events or helping events were coded. Interrater agreement was excellent (κ = 1) in both tasks.

### Results

**Figure 2:**
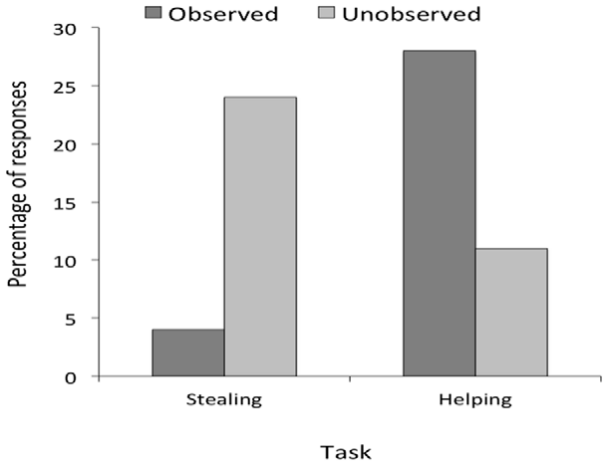
Results of the child study. Mean percentage of responses as a function of task and condition in Experiment 1.

Figure 2 shows the results of both the stealing task and helping task. In the stealing task, subjects stole in 4% of all cases in the observed and in 24% of all cases in the unobserved condition. A Mann-Whitney *U* exact test found this difference to be statistically reliable (*U*(24,24) = 223.5, p = .02, one-tailed). In the helping task, subjects helped in 11% of all cases in the unobserved and in 28% of all cases in the observed condition. A Mann-Whitney *U* exact test found a trend toward a significant difference (*U*(24,24) = 228, p = .07, one-tailed).

### Discussion

Experiment 1 found that children as young as 5 years of age engage in self-reputational behavior. Thus, children stole less and tended to help more in the observed compared to the unobserved condition. The fact that the stealing result was statistically reliable while the helping result was only a trend is likely due to the different norms and rules involved in the two tasks. Specifically, self-reputational behavior in the helping task would involve an understanding of the social norm of helping someone in need. In the stealing task, on the other hand, such behavior would involve understanding the social rule that stealing is not allowed. It thus seems plausible that young children are more aware of the negative reputational consequences of breaking a salient social rule as opposed to a rather complex social norm involving an assessment of need.

Importantly, in our experimental design the observer was an unfamiliar child and the recipient was absent, thus effectively ruling out explanations based on the familiarity of either observer or recipient and the fear of authority (in the case of an adult observer).

## Study 2: Chimpanzees

In order to explore the evolutionary foundations of this human impression management behavior, we ran a similar study with humans' nearest primate relatives, chimpanzees.

### Method

#### Ethics Statement

Research at the WKPRC was performed in accordance with the recommendations of the Weatherall report “The use of non-human primates in research”. Groups of apes were housed in semi-natural indoor and outdoor enclosures with regular feedings, daily enrichment and water ad lib. Subjects voluntarily participated in the study and were never food or water deprived. Research was conducted in the sleeping and/or observation rooms. No medical, toxicological or neurobiological research of any kind is conducted at the WKPRC. Research was non-invasive and strictly adhered to the legal requirements of Germany. The full procedure of the study was approved by the Max Planck Institute for Evolutionary Anthropology Ethics Committee (members of the committee are Prof. M. Tomasello, Dr. J. Call, Dr. D. Hanus, veterinarian Dr. A. Bernhard, head keeper F. Schellhardt and assistant head keeper M. Lohse). Animal husbandry and research comply with the “EAZA Minimum Standards for the Accommodation and Care of Animals in Zoos and Aquaria”, the “WAZA Ethical Guidelines for the Conduct of Research on Animals by Zoos and Aquariums” and the “Guidelines for the Treatment of Animals in Behavioral Research and Teaching” of the Association for the Study of Animal Behavior (ASAB). IRB approval was not necessary because no special permission for the use of animals in purely behavioral or observational studies is required in Germany. Further information on this legislature can be found in paragraphs 7.1, 7.2 and 8.1 of the German Protection of Animals Act (“Tierschutzgesetz”).

#### Participants

Fourteen chimpanzees (9 females and 5 males), ranging in age from 6 to 33 years (*M* = 20 years), participated in Study 2. A low-ranking female was chosen as recipient (age  = 9 years). A high-ranking male acted as observer (age  = 33 years). This was thought to generate increased reputational concern, as chimpanzees' fitness increases with strategic partnerships with dominant individuals (Silk, 2007). The recipient was present in both conditions, observed and unobserved, but visually hidden by occluders. The chimpanzees were socially housed at the Wolfgang Köhler Primate Research Center in Leipzig, Germany.

#### Materials

**Figure 3:**
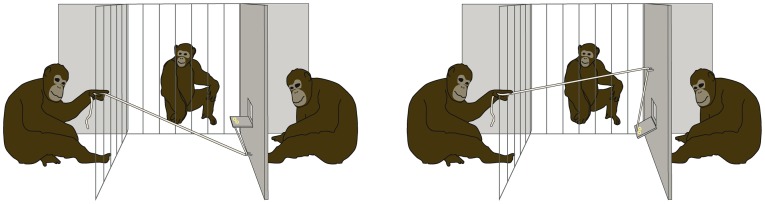
Setup of the chimpanzee study. Illustration of the experimental setup for chimpanzees, viewed from the experimenter's point of view. The observed condition (pictured here) consisted of three different roles, subject (left), observer (middle) and receiver (right). In the stealing task (left), subjects could steal food from the receiver by collapsing the food platform. In the helping task (left), subjects could give food to the recipient, which they couldn't obtain otherwise.

For both the stealing and the helping tasks, subjects had the possibility of pulling a rope from their room. The rope was attached to a wooden platform, which could only be accessed from the other, recipient's, room (Figure 3). The platform was in different positions for the two tasks. In the stealing task, the platform initially was in a position such that the recipient could easily access the food upon it. Pulling the rope moved the food to a position where no one could reach it, whereas refraining from pulling left the food available to the recipient. In the helping task, the platform and food were initially out of the recipient's reach. Here, pulling the rope moved the food to within her reach, whereas refraining from pulling left the food out of the recipient's reach. In both tasks, food was a mix of grapes, small pellets, and raisins.

#### Procedure and Design

We employed a within-subjects design across task and condition. Thus, subjects participated in both tasks, helping and stealing, and both conditions, observed and unobserved. Half of subjects started with the helping task, the other half with the stealing task, and the order of conditions was counterbalanced across subjects as well. Each session consisted of 4 blocked trials of the given condition. Before the test phase, each chimpanzee was introduced to the apparatus to ensure an understanding of its mechanisms. Testing took place in the chimpanzees' sleeping area.

In the stealing task, the introductory phase consisted of an “open door” and a “closed door” situation with three trials each. In the open door situation, doors between the testing units were open and subjects could move freely within the three rooms. Because food was accessible only from the room away from the rope, subjects had to inhibit pulling the rope (not steal) in order to gain access to the food in the other room (and pulling the rope was irreversible). Only when subjects had reached the criterion of accessing the food 3 times in a row (within a maximum of 8 trials) did they pass from the “open door” to the “closed door” situation. Nine subjects passed the criterion within the first 4 trials; all subjects passed the criterion within 8 trials.

In the “closed door” situation, the doors between the rooms were closed, as they would be in the test situation. Thus, subjects learnt that they did not have access to the food, independent of their decision to pull the rope or not. Subjects were expected to pull the rope in the “closed door” situation only infrequently, as it led to no rewarding result. Indeed, in 3 trials only one subject pulled the rope twice and two subjects once, thus showing an understanding of the situation.

In the helping task, the introductory phase also consisted of an “open door” and a “closed door” situation of 3 trials each. In the “open door” situation subjects learned that if they pulled the rope in one room the food could be accessed from the opposite room. Subjects moved from the “open door” to the “closed door” condition only after they passed the criterion of accessing the food 3 times in a row within a maximum of 8 trials. Twelve subjects passed the criterion within the first 4 trials, the remaining 2 subjects within 8 trials.

In the “closed door” situation, the doors between the rooms were closed, as they would be in the test situation. Again, as in the stealing condition, subjects learnt that they did not have access to the food, independent of their decision to pull the rope or not. In the “closed door” condition, pulling frequency declined over the course of 3 trials.

The general procedure for testing was the same for both tasks. On testing day, each subject first underwent a refresher that consisted of one trial of both the “open door” and “closed door” situations. During testing, depending on condition, the observer was either present or absent. Once all relevant apes were positioned in their rooms, in both conditions of both tasks, Experimenter 1 attracted the subject away from the apparatus while Experimenter 2 placed food on the platform and extended the rope into the subject's room. Both experimenters then left the area. After 60 seconds, Experimenter 1 returned to the room to prepare for the next trial.

#### Coding and dependent measure

All trials were videotaped with 4 cameras and coded by the first author. A research assistant, unaware of the study design and hypothesis, independently coded 25% of all trials. Number of stealing events or helping events were coded. Interrater agreement (κ) was excellent at.9 (stealing) and 1 (helping).

### Results

**Figure 4:**
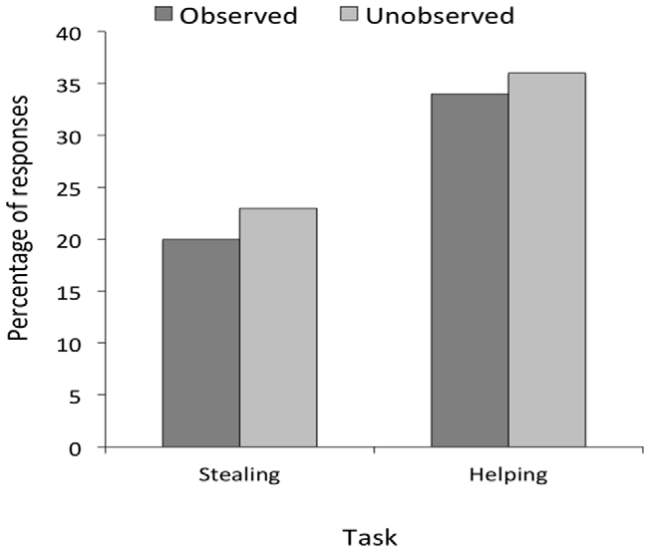
Results of the chimpanzee study. Mean percentage of responses as a function of task and condition in Experiment 2.

As seen in figure 4, responses in the observed conditions were very similar to responses in the unobserved conditions in both tasks. Specifically, in the helping task, subjects helped in 34% of all cases in the observed condition and 36% of all cases in the unobserved condition (Wilcoxon Signed Ranks Test, *z* = −.272, p = .47, one-tailed). In the stealing task, subjects stole in 20% of all cases in the observed condition and in 23% of all cases in the unobserved condition (Wilcoxon Signed Ranks Test, *z* = −.136, p = .50, one-tailed).

### Discussion

In this experiment chimpanzees showed no evidence of any self-presentational (impression management) behaviors. Subjects showed a constant helping and stealing rate, independent of condition. One might argue that the chimpanzees did not fully understand the apparatus, but the results from the introductory phase of both tasks demonstrate that they did. One might also worry that somehow subjects thought that the recipient was observing them in the unobserved conditions (unlike the child study where children could be told of a mythical other child, the chimpanzees had to know of a real recipient). But we blocked their view so they could not see each other (though they might hear each other), and, moreover, the recipient was a subordinate individual, whose reputational judgment should be less important. Crucially, in the observed conditions an alpha-male was watching, in full view of the subject, which should evoke a much greater reputational concern in subjects capable of such concern.

## General Discussion

In the current study, we found impression management (self-presentational) behavior in 5-year-old children, the youngest age found to date. This finding effectively falsifies the hypothesis [14], [16] that children this young, though cognitively capable, are not concerned with the impression they are making on others. Our findings were a bit stronger for the stealing task than for the helping task, perhaps because a reputation as a thief is worse than a reputation as a person who does not help when she could (or who does help when she can). The fact that the dimension of reputation at issue here was cooperation (as opposed to, for instance, a reputation for competence) fits well with current theories suggesting that, among primates, humans are especially cooperative [23] and that cooperation is especially important in human societies [24].

In contrast, the chimpanzees in our study did not behave differently in either the helping or the stealing task when they were being watched by a dominant conspecific. We had hypothesized that the chimpanzees might have different reputational concerns than the children, and so actually steal more often when being watched to increase their reputation for dominant behavior. But we did not observe this pattern of behavior either. It is of course possible that we might find a concern for reputation in chimpanzees in other situations, for example, ones in which they would experience an immediate negative consequence of a negative judgment by others. But the fact that the observer was a high-ranking male should have made the potential consequences, for example, of stealing from another, relatively salient [25]. It is also possible that chimpanzees would show a concern for a reputation for being competent, or some other trait, as opposed to being cooperative or non-cooperative.

Regarding the design of the two tasks, stealing and helping, two differences to Study 1 were introduced in Study 2. One, during the stealing task, chimpanzees could not obtain the stolen food. This feature was introduced in Study 2 as it was expected that stealing frequency would have been too high, i.e. at ceiling, if chimpanzee subjects would have been given the possibility of stealing and eating the food from a lower-ranking individual [26]. Two, during the helping task, chimpanzees did not initially possess the food which they could then give to a conspecific. This was introduced to reduce the cost associated with the helping behavior and prevent a possible “floor-effect” as a lot of research supports the argument that chimpanzees do not engage in voluntary food sharing, especially not with low-ranking individuals [26]–[28]. The relatively low-cost nature of helping in Study 2 is also thought to explain the fact that chimpanzees on average helped more than the children in Study 1. This difference in design, however, cannot explain the observed pattern of results.

The explanation for the observed species difference might be either cognitive or motivational. Tomasello [29] argues that a variety of evidence shows that chimpanzees cannot engage in the kind of recursive mindreading (understanding that the other is evaluating my intentional states) that would seem to be necessary for strategic self-presentational behavior. Whereas chimpanzees are capable of some theory of mind abilities, they seem to lack the capacity for such meta-representations [30]. Children on the other hand have routinely been shown to pass meta-representation tests, such as false belief tests, from at least the age of five onwards [19]. This argument also fits nicely with recent results from social neuroscience. Izuma and colleagues [31], [32] have shown that the same area that is involved in meta-representations, the medial prefrontal cortex (mPFC), also plays a crucial function in reputation management.

The motivational possibility is that while chimpanzees for example help both conspecifics and humans [33], [34], the social structure of chimpanzees is such that they know that the other is evaluating them but they do not care. This is possible, but one would certainly think that, especially in the case of stealing, a high-ranking male should give lower ranking individuals pause – but this still might be on the level of behavior and not reputation. So, in all, although further evidence from other domains is needed, our inclination is to support the hypothesis that both cognitive and motivational factors are responsible.

A further interesting point relates to the conceptual relationship between reputation management and punishment. Regarding Study 1, one could argue that children's behavior in the observed stealing condition can be explained more plausibly by avoidance of punishment. However, we believe that the use of a peer observer (instead of an authority figure) makes that explanation relatively unlikely. In addition, results from the helping task, showing a tendency in 5-year olds to help more in an observed relative to an unobserved condition, are not explicable by an avoidance of punishment strategy. Furthermore, especially in development, an avoidance of punishment strategy and an emerging sense of one's reputation might interact in interesting ways. A recent study by Banerjee and colleagues [35] explicitly highlights the crucial role of rule violations as key contexts for children's learning about public identity. We believe that the relationship between punishment and reputation provides an interesting avenue for future theoretical and empirical research.

Besides highlighting the crucial role of punishment [36], [37], current theories of the evolution of human cooperation stress the role of social selection and partner choice [38], [39]. Thus, individuals who attempt to dominate non-cooperatively are subject to coalitions of counter-dominance or reputation-killing gossip [40], [41]. In addition, when individuals depend on one another in cooperative activities they must keep up their good reputation in order to keep being chosen to participate [38], in which case concern for self-reputation serves as a counterpoint to so-called “cheater detection”. If others are choosing their partners and are constantly vigilant to exclude cheaters, then I must appear to be cooperative – and the best way to appear to be cooperative is to actually be cooperative. In this regard, recent results showing that the cleaner fish *Labroides dimidiatus* behaves more cooperatively in the presence of an audience are especially interesting [42].

In terms of ontogeny, we of course do not believe that the beginnings of a concern for self-reputation that we have observed here are anything like the end of the story. Experiences in peer groups during the elementary school years equip children with a much deeper understanding of reputational mechanisms and how they work. For example, it is probably not before school age that gossiping becomes an integral part of reputation formation [43], [44]. And, of course, during adolescence a concern with self-reputation and attempts at impression management reach their apex, as being accepted by various groups and subgroups becomes critical to social well-being. In any case, the current study demonstrates that this relatively protracted developmental process begins already during the preschool period.

When individuals are concerned about their self-reputation, then, they are motivated to cooperate, especially when others are observing, and observers can have this effect at very low cost to themselves, that is, without the threat of direct punishment but only the threat of a bad opinion.
